# Lipoprotein(a) and the Risk for Recurrent Ischemic Stroke Events∗

**DOI:** 10.1016/j.jacadv.2023.100559

**Published:** 2023-08-21

**Authors:** Robert S. Rosenson, Lisandro D. Colantonio

**Affiliations:** a Metabolism and Lipoprotein Unit, Mount Sinai Heart, Icahn School of Medicine at Mount Sinai, New York, New York, USA; b Department of Epidemiology, University of Alabama at Birmingham, Birmingham, Alabama, USA

**Keywords:** coronary heart disease, lipoprotein(a), low-density lipoprotein, statins, stroke

**Figure:**
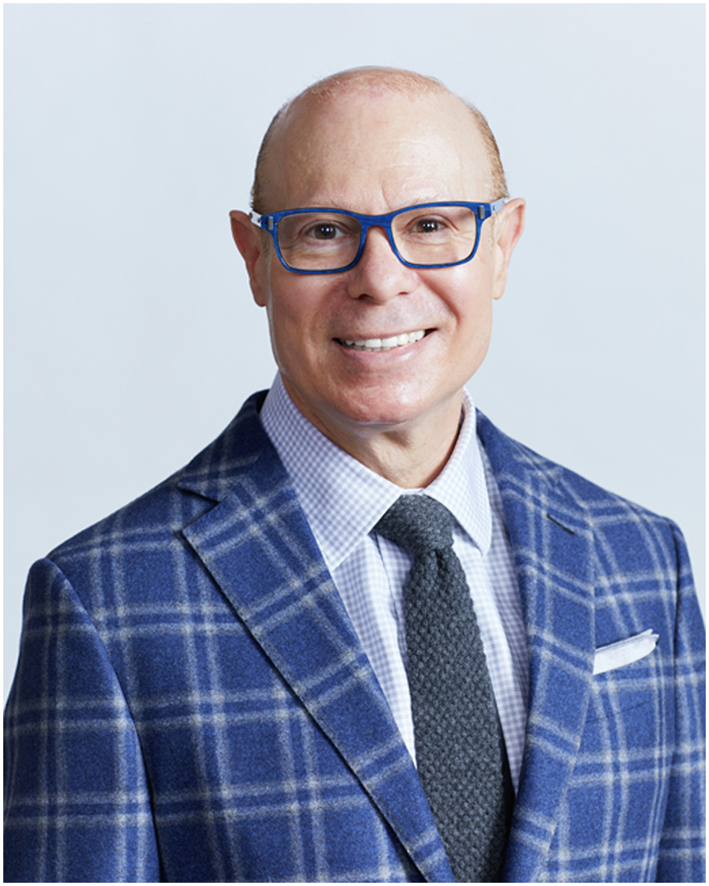

Stroke is the leading cause of disability worldwide and the fifth most common cause of death in the United States.^1^ Among stroke events, 23% are recurrent strokes.^1^ Secondary prevention of ischemic stroke, the most frequent type of stroke, includes the lifestyle interventions of smoking cessation, reducing, or eliminating alcohol consumption, a healthy diet with salt restriction, weight reduction in overweight patients, and regular exercise. Treatment with antiplatelet therapy, blood pressure control, and low-density lipoprotein cholesterol (LDL-C) lowering with high-intensity statin therapy is also recommended.

Evidence for LDL-C lowering in stroke survivors is based on 2 pivotal trials of high-intensity statin therapy.^2^^,^^3^ The SPARCL (Stroke Prevention by Aggressive Reduction in Cholesterol Levels) trial was the first to demonstrate that statin therapy reduced the risk of recurrent stroke.^2^ The trial included 4,731 patients with a history of stroke or transient ischemic attack (TIA) and LDL-C 100 to 190 mg/dL who were randomized to receive atorvastatin 80 mg daily or placebo. After a median 4.9 years of follow-up, treatment with atorvastatin reduced the risk of fatal or nonfatal stroke (11.2% vs 13.1%; adjusted HR: 0.84; 95% CI: 0.71-0.99), all coronary heart disease events (5.2% vs 8.6%; HR: 0.58; 95% CI: 0.46-0.73), and all cardiovascular events (22.4% vs 29.0%; HR: 0.74; 95% CI: 0.66-0.83). The TST (Treat Stroke to Target) trial investigated prespecified LDL-C targets in patients with a recent ischemic stroke or TIA who had LDL-C ≥70 mg/dL if taking a statin or ≥100 mg/dL if not taking a statin.^3^ The trial included 2,860 patients were randomly assigned to a lower target LDL-C <70 mg/dL or to a higher target LDL-C of 90 to 110 mg/dL. The composite primary end point of major cardiovascular events (ischemic stroke, myocardial infarction, new symptoms leading to urgent coronary or carotid revascularization, or cardiovascular death) was reduced in the lower target LDL-C group compared with the higher target group (8.5% vs 10.9%; adjusted HR: 0.78; 95% CI: 0.61-0.98).

Stroke reduction was also reported in clinical trials with PCSK9 monoclonal antibodies among adults receiving statin therapy. The FOURIER (Further Cardiovascular Outcomes Research with PCSK9 Inhibition in Subjects with Elevated Risk) trial reported that treatment with evolocumab vs placebo reduced ischemic strokes (1.2% vs 1.6%; adjusted HR: 0.75; 95% CI: 0.62-0.92).^4^ Treatment with alirocumab also reduced the risk of fatal or nonfatal stroke vs placebo (1.2% vs 1.6%; adjusted HR: 0.73; 95% CI: 0.57-0.93) in the ODYSSEY (Outcomes Alirocumab and Cardiovascular Outcomes after Acute Coronary Syndrome).^5^ These studies provide evidence that the risk of recurrent stroke can be reduced by lowering LDL-C <70 mg/dL with the use of PCSK9 monoclonal antibodies added to statin therapy.

LDL-C measurements encompass the cholesterol content of lipoprotein(a) [Lp(a)], which has itself emerged a risk marker for cardiovascular events.^6^ In this issue of *JACC: Advances*, the SPARCL investigators examined the contribution of Lp(a) molar concentration and apo(a) isoform sizes with subsequent cerebrovascular and cardiovascular events in stroke/TIA survivors.^7^ This analysis included 2,814 SPARCL participants. Multivariate-adjusted HR was calculated for the highest Lp(a) quartile (≥84.0 nmol/L) vs the lowest Lp(a) quartiles (<84.0 nmol/L). Among randomized patients, there was no association between Lp(a) molar concentrations or apo(a) isoform sizes and the risk of recurrent stroke or cerebrovascular events. In the atorvastatin treatment group, high Lp(a) molar concentrations and apo(a) isoforms were associated with an increased risk of coronary events (HR: 1.607; [95% CI: 1.007-2.563] and HR: 2.052 [95% CI: 1.303-3.232], respectively). No associations were observed in the placebo group. In summary, this analysis of SPARCL confirms previous studies that high Lp(a) concentrations and apo(a) isoform sizes are risk factors for coronary events. The lack of association of Lp(a) with recurrent stroke events in SPARCL needs to be interpreted with caution. A recent Mendelian randomization study reported that genetically predicted Lp(a) was associated with a marginal increase in ischemic stroke (OR: 1.004; 95% CI: 1.001-1.007).^8^ Also, the association for recurrent stroke events may be weaker than for incident stroke.

Several limitations of this study warrant comment. The study included patients with an elevated LDL-C independent of the Lp(a) concentration, whereas most studies report that the Lp(a) contribution to cardiovascular risk occurs at higher levels than observed in the upper quartile distribution in SPARCL.^9^ The diagnosis of stroke was clinically based and did not require neuroimaging. This may have resulted in inclusion of nonischemic etiologies. There were different associations between Lp(a) and the risk for coronary events between the atorvastatin and placebo-treated patients, which is perplexing. This finding contrasts with our prior analysis of REGARDS (REasons for Geographic and Racial Differences in Stroke) study participants in which the risk for coronary events associated with Lp(a) was similar between statin and nonstatin users.^10^ A possible explanation for the SPARCL results is residual confounding. Specifically, hypertension prevalence was higher in the upper vs lower Lp(a) quartiles of the atorvastatin group, which may have contributed to the higher coronary risk. In contrast, hypertension was less prevalent in the upper vs lower Lp(a) quartiles of the placebo group, which may have contributed to a lower coronary risk. Although the authors adjusted for hypertension, there may be still imbalance in blood pressure control or other unmeasured confounders. Finally, it has been reported that Lp(a)-associated risk is influenced by systemic inflammation^11^; however, measures of interleukin-6 or C-reactive protein were unavailable in this trial.

Determination of whether lowering Lp(a) reduces stroke risk awaits completion of the HORIZON (Randomized Double-blind, Placebo-controlled, Multicenter Trial Assessing the Impact of Lipoprotein (a) Lowering With Pelacarsen [TQJ230] on Major Cardiovascular Events in Patients With Established Cardiovascular Disease; NCT04023552). This trial enrolled very high-risk patients with either ischemic stroke, myocardial infarction, or symptomatic peripheral artery disease. In contrast, the OCEAN(a) Outcomes Trial (Olpasiran Trials of Cardiovascular Events and Lipoprotein(a) Reduction; NCT05581303), another trial of an Lp(a)-lowering therapy, is enrolling patients with coronary heart disease.

Until completion of clinical trials with Lp(a)-lowering therapies, we support screening for Lp(a) molar concentrations in adults with a stroke after resolution of the acute phase response as recommended by the American Heart Association.^6^ Interventions to reduce the risk for recurrent stroke events in these patients should include lifestyle modifications, control of cardiovascular risk factors, and lowering LDL-C <55 mg/dL with a high-intensity statin.^12^ For stroke patients with an LDL-C ≥55 mg/dL despite taking maximally tolerated statin therapy who have an Lp(a) level >125 nmol/L, PCSK9 monoclonal antibodies could be considered based on subgroup analyses of FOURIER and ODYSSEY Outcomes that reported this therapy reduces the risk for major cardiovascular events and this risk reduction was larger in patients with high vs low levels of Lp(a) (Figure 1).^12^ In patients with LDL-C ≥55 mg/dL, Lp(a) >125 nmol/L, and LDL-C <25% above the target, ezetimibe is an option.

**Figure 1:**
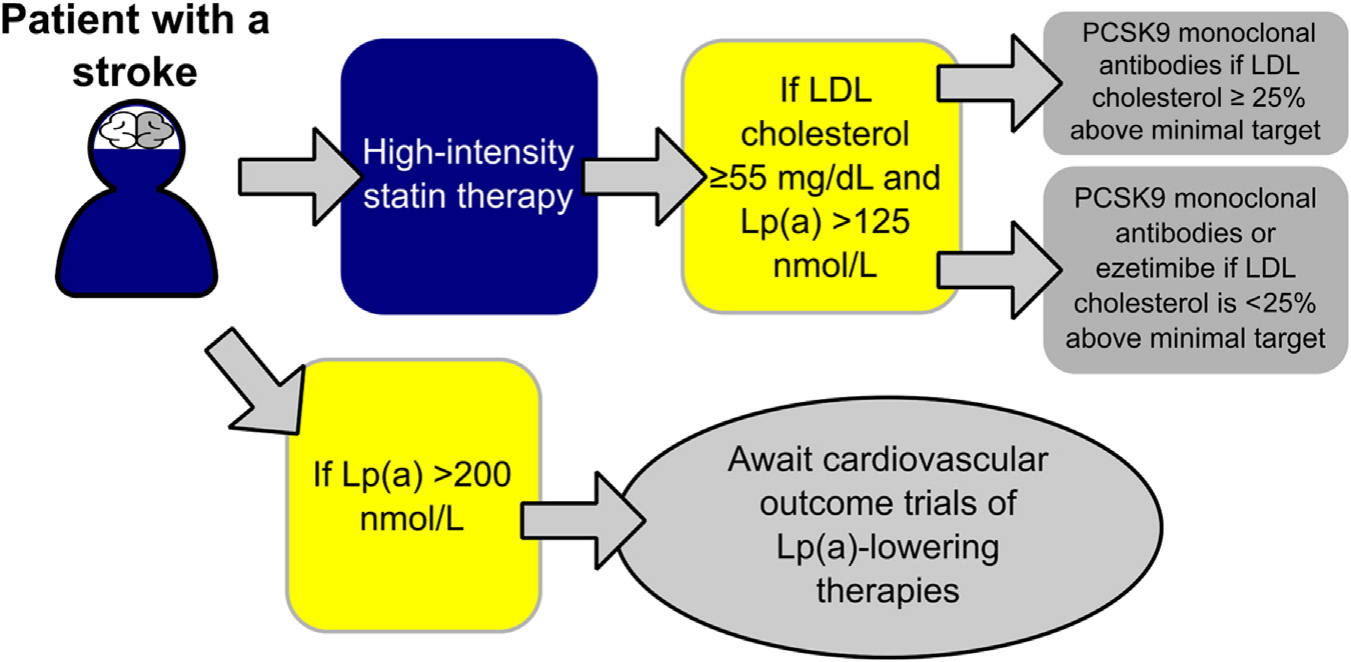
**Translating SPARCL-Lp(a) into Clinical Practice** Among SPARCL patients taking atorvastatin 80 mg daily, high vs low Lp(a) levels was associated with a lower risk of coronary heart disease events but not stroke events. Until completion of randomized clinical trials with selective Lp(a) lowering therapies, we advocate a PCSK9 monoclonal antibody inhibitor to lower LDL-C <55 mg/dL in patients with high Lp(a) levels, particularly when the LDL-C level is >25% above targeted levels. LDL-C = low-density lipoprotein cholesterol.

## Funding support and author disclosures

Dr Colantonio has received research support from Amgen, Inc. The author has reported that they have no relationships relevant to the contents of this paper to disclose.
